# A Unified Anatomy Ontology of the Vertebrate Skeletal System

**DOI:** 10.1371/journal.pone.0051070

**Published:** 2012-12-10

**Authors:** Wasila M. Dahdul, James P. Balhoff, David C. Blackburn, Alexander D. Diehl, Melissa A. Haendel, Brian K. Hall, Hilmar Lapp, John G. Lundberg, Christopher J. Mungall, Martin Ringwald, Erik Segerdell, Ceri E. Van Slyke, Matthew K. Vickaryous, Monte Westerfield, Paula M. Mabee

**Affiliations:** 1 Department of Biology, University of South Dakota, Vermillion, South Dakota, United States of America; 2 National Evolutionary Synthesis Center, Durham, North Carolina, United States of America; 3 Department of Biology, University of North Carolina, Chapel Hill, North Carolina, United States of America; 4 Department of Vertebrate Zoology and Anthropology, California Academy of Sciences, San Francisco, California, United States of America; 5 The Jacobs Neurological Institute, University at Buffalo, Buffalo, New York, United States of America; 6 Oregon Health and Science University, Portland, Oregon, United States of America; 7 Department of Biology, Dalhousie University, Halifax, Nova Scotia, Canada; 8 Department of Ichthyology, The Academy of Natural Sciences, Philadelphia, Pennsylvania, United States of America; 9 Genomics Division, Lawrence Berkeley National Laboratory, Berkeley, California, United States of America; 10 The Jackson Laboratory, Bar Harbor, Maine, United States of America; 11 Zebrafish Information Network, University of Oregon, Eugene, Oregon, United States of America; 12 Department of Biomedical Sciences, Ontario Veterinary College, University of Guelph, Guelph, Ontario, Canada; 13 Institute of Neuroscience, University of Oregon, Eugene, Oregon, United States of America; University of Lausanne, Switzerland

## Abstract

The skeleton is of fundamental importance in research in comparative vertebrate morphology, paleontology, biomechanics, developmental biology, and systematics. Motivated by research questions that require computational access to and comparative reasoning across the diverse skeletal phenotypes of vertebrates, we developed a module of anatomical concepts for the skeletal system, the Vertebrate Skeletal Anatomy Ontology (VSAO), to accommodate and unify the existing skeletal terminologies for the species-specific (mouse, the frog *Xenopus*, zebrafish) and multispecies (teleost, amphibian) vertebrate anatomy ontologies. Previous differences between these terminologies prevented even simple queries across databases pertaining to vertebrate morphology. This module of upper-level and specific skeletal terms currently includes 223 defined terms and 179 synonyms that integrate skeletal cells, tissues, biological processes, organs (skeletal elements such as bones and cartilages), and subdivisions of the skeletal system. The VSAO is designed to integrate with other ontologies, including the Common Anatomy Reference Ontology (CARO), Gene Ontology (GO), Uberon, and Cell Ontology (CL), and it is freely available to the community to be updated with additional terms required for research. Its structure accommodates anatomical variation among vertebrate species in development, structure, and composition. Annotation of diverse vertebrate phenotypes with this ontology will enable novel inquiries across the full spectrum of phenotypic diversity.

## Introduction

In the discipline of comparative morphology [1], phenotypic diversity is described in free text in a variety of ways, including detailed anatomical studies, descriptions of new species, and characters used in phylogenetic analyses. However, it is often difficult to compare phenotypes across taxa because of the different terminologies used in these descriptions. Researchers studying different anatomical regions, different taxa, or working within different biological specialties often have dissimilar terminologies [2]. Furthermore, even when the same term is used, identifying publications that analyze the same structure is not trivial, and combining character matrices across studies is an even larger hurdle [3]. If phenotypic diversity were represented in a common and computable manner, one would be better able to explore the wealth of data available across a broad range of anatomy, development, and taxa and also to relate this information to different domains of biological knowledge such as genomics, comparative embryology, and functional morphology [4], [5]. By grappling with phenotypic diversity in a structured and formal way, novel inquiries can be made across organismal phenotypic diversity, including evolved natural phenotypes and the mutant phenotypes of model systems.

This synthesis and discovery can be made feasible through the use of shared ontologies [6], [7]. An ontology is a structured, controlled vocabulary in which the terms and the relationships between the terms are defined using formal logic. It represents the knowledge of a discipline in a format that can be understood both by humans and by machines for computational inference. Ontology-based searches differ from keyword and text searches because they allow one to retrieve groups of related terms rather than only direct text matches of search terms. The reason for improved retrieval is that one can exploit the logical definitions [8], [9] and relations across terms and thereby infer additional information. Using an anatomy ontology with logical links to development and a database of ontology-based annotations to multiple species, for example, one might search for ‘intramembranous ossification’ and return frog ‘frontoparietal bone’ because it develops using this mode of ossification. One would also return chick ‘tibia’, an endochondral bone, because it also undergoes intramembranous ossification along the midshaft [10]. Furthermore, even the simple use of synonyms facilitates retrieval; for example, a user searching on ‘skull’ would retrieve data tagged with ‘cranium’. Thus, an ontology can support grouping and comparison of data in significant ways by leveraging the logical relationships among concepts.

Ontologies can be used for standardizing terminology within disciplines and for clarifying and improving communication across domains. Most importantly, ontologies can be used to bring together disparate data in a logically consistent manner. Many anatomy ontologies are restricted to model organisms and are used for annotating gene expression and resulting phenotypes: for example if *sonic hedgehog a* is not expressed in the neural tube of the zebrafish, the anterior neural tube is malformed [11]. Recently, the evolutionary biology community has also begun to use anatomy ontologies because they provide a structured representation for comparative morphology and the potential to link comparative morphological data to the wealth of genomic, anatomical, and phenotype data available in model organism databases [12], [13], [14], [15], [16]. However, model organism and taxon-specific anatomy ontologies have been largely developed semi-independently within their specific communities. As a result, the terminological subclass hierarchies of anatomical parts developed by different communities are frequently divergent. This poses significant obstacles to integrating data across species or projects. The resulting confusion can be remedied by consensus among workers from different disciplines, such as by bringing representatives from various domains together to agree on at least a common upper-level ontology, or by developing a bridging ontology that can be used for reasoning [17].

Motivated by comparative research questions that require reasoning across the taxonomic and phenotypic diversity of vertebrate skeletal morphologies at different biological scales, we sought a higher-level representation of skeletal anatomy that reconciles currently existing species-specific and multispecies ontological representations of the skeletal system (Table 1). To this end, we, a group of anatomy experts and ontologists, worked together to develop a module of high-level anatomy ontology concepts that unify more specific terms for the skeletal system. This module, which we call the Vertebrate Skeletal Anatomy Ontology (VSAO), integrates terms for cells, tissues, biological processes, organs (skeletal elements such as bones and cartilages), and subdivisions of the skeletal system, thus enabling novel queries and computation across different levels of granularity and taxa. The upper-level skeletal terms in the VSAO can easily integrate terms for more specific structures and tissue types, including structures found in taxa that are not currently covered by existing anatomy ontologies. For example, placoderms, a group of extinct fossil fishes, possess a ‘scapular complex’, a cluster of dermal bones represented in VSAO as a type of ‘skeletal subdivision’ that is part of the pectoral girdle [18].

**Table 1:** Vertebrate anatomy ontologies and others formally related to VSAO (*applicable to multiple species).

Abbreviation	Ontology name	Taxon	Reference	Associated database or source (URL)
AAO	Amphibian Anatomy Ontology*	Amphibia	[16]	http://obofoundry.org/cgi-bin/detail.cgi?id=amphibian_anatomy
CARO	Common Anatomy Reference Ontology*		[17]	http://code.google.com/p/caro2/
CL	Cell Ontology*		[25], [26]	http://cellontology.org/
FMA	Foundational Model of Anatomy	Human, *Homo sapiens*	[38]	http://sig.biostr.washington.edu/projects/fm/
GO	Gene Ontology*		[27]	http://www.geneontology.org/
MA	Mouse Adult Gross Anatomy	Mouse, *Mus musculus*	[48]	http://www.informatics.jax.org/searches/AMA_form.shtml
PATO	Phenotype and Trait Ontology*		[30]	http://obofoundry.org/wiki/index.php/PATO:Main_Page
TAO	Teleost Anatomy Ontology*	Teleostei	[13]	http://phenoscape.org/wiki/Teleost_Anatomy_Ontology
Uberon	Uber Anatomy Ontology*	Metazoa	[28]	http://obofoundry.org/wiki/index.php/UBERON:Main_Page
XAO	*Xenopus* Anatomy Ontology	African clawed frogs, *Xenopus laevis; X. tropicalis*)	[35]	http://www.xenbase.org
ZFA	Zebrafish Anatomy Ontology	Zebrafish, *Danio rerio*	[49]	http://www.zfin.org

Ontology files can be downloaded from the Open Biological and Biomedical Ontologies Foundry (http://obofoundry.org/).

Rather than representing one strict classification of skeletal anatomy, the goal of developing these concepts was to accommodate the breadth of ways that biologists classify skeletal entities. The VSAO set of high-level skeletal system concepts will be a valuable resource to the fields of comparative morphology, development and genetics because of its integrative goal to unify existing vertebrate ontologies, thus enabling queries of disparate data sets across taxa, experimental studies, phylogenetic analyses, and genomics.

## Methods

### Content

Refinement and development of an integrated upper-level term set for the skeletal system was motivated by the recognition that the existing vertebrate anatomy ontologies for single and multiple species (Table 1) differ in their representations of the skeletal system, which prevents effective reasoning across associated databases. We took an iterative approach by creating a new set of high-level anatomical concepts *de novo*, comparing it with the existing high-level hierarchies of the various vertebrate anatomy ontologies, and making revisions accordingly. We focused on unification, standardization, and expansion of terms and relations associated with the skeletal system. The VSAO module mainly includes high-level terms such as ‘bone element’ and ‘bone tissue’ that unify more specific terms, but it also includes terms for specific bones and cartilages including some that are present in vertebrates but not covered by other subsumed vertebrate anatomy ontologies (e.g., the placoderm ‘scapular complex’). The initial version of VSAO that contains the 139 high-level terms, 62 synonyms, and relationships discussed by the coauthors of this paper at a workshop is available for download [19]. The version of VSAO described here has grown to include 223 terms and 179 synonyms, excluding 50 terms imported from CARO, and is available for download in OBO and OWL formats [20] and can be browsed through the NCBO BioPortal [21] and OntoBee [22]. Both versions are deposited in the Dryad Repository [23]. VSAO terms are given both text and logical definitions with attribution including but not limited to a reference ID to the workshop [24]. Terms added or proposed to Cell Ontology (CL) [25], [26] and Gene Ontology (GO) [27] are also referenced to this workshop [24].

### Ontology Construction Principles

Ontologies are referred to herein using their formal namespace abbreviations (Table 1). The development of the VSAO followed the principles of the Open Biological and Biomedical Ontologies Foundry (http://obofoundry.org). The VSAO is freely available, maintained in a version control system to record and make accessible the development history, and is accessible to the community in both OBO and OWL syntax. Terms consist of a unique identifier (‘VSAO’) followed by a stable, unique seven digit numerical code associated with a label, text definition, and synonyms that, unlike the identifier, can be modified. Identifiers for terms no longer considered valid are marked as obsolete rather than deleted from the ontology, and the identifier is preserved. We are working towards the OBO Foundry principle of maintaining clearly delineated content in VSAO with the goal of being orthogonal (non-overlapping and integrated) with other ontologies in the OBO Foundry. Integration of VSAO and other well established anatomy ontologies for vertebrate species into the Uber Anatomy Ontology (Uberon) [28] will advance this admittedly difficult goal [29].

The VSAO includes terms from several species-independent ontologies (Table 1), including the Common Anatomy Reference Ontology (CARO) [17], which provides high-level classes that link together different levels of anatomical organization; the Gene Ontology (GO) [27], which provides biological process classes involved in development and function of the skeletal system; the Cell Ontology (CL) [25], [26], which provides cell types of the skeletal system; and the Phenotype and Trait Ontology (PATO) [30], which provides quality descriptors (for example, ‘ossified’) used in logical definitions. As terms relevant to the skeletal system are added to these ontologies, they will be connected to the VSAO. Because anatomical terms must be accurately connected across the various levels of biological organization and across different axes of classification for meaningful reasoning, we related terms to one another through logical relationships including *is_a*, *part_of*, and *develops_from*, which are relationships commonly used in anatomy ontologies [31]. The relationships are formally defined in Smith et al. [29] and in the Relations Ontology (RO; http://obofoundry.org). RO:*is_a* is semantically the same as owl:subClassOf (http://www.geneontology.org/GO.format.obo-1_4.shtml). Classes are denoted in single quotes herein (e.g., ‘bone tissue’) and relations are shown in italics (e.g., *part_of*). Gross organism subdivision terms such as ‘fin’ are cross-referenced to Uberon [28]. Anatomical classes in the VSAO are defined using structural, positional, functional and developmental criteria. The VSAO strictly describes anatomy rather than the distribution of skeletal classes across organismal clades. The distribution of skeletal features across species can be annotated using a taxonomy ontology in a database of phenotype statements, an endeavor that will be driven by the research demands of different communities (e.g., kb.phenoscape.org). The VSAO makes no explicit assertions regarding homology of skeletal entities across taxa. Our premise is that homology should be asserted outside the ontology. Homology between structures across taxa may thus be asserted by users, along with annotations of evidence and attribution, which allows different hypotheses of homology to be explored [13].

Taxon-specific vertebrate ontologies vary in their formal relationships to the VSAO. For example, the Teleost Anatomy Ontology (TAO) [13] imports the entirety of the VSAO rather than duplicating terms; therefore, a teleost TAO: ‘maxilla’ *is_a* vertebrate VSAO: ‘dermal bone’ (TAO can be browsed in BioPortal [32] and Ontobee [33]; the TAO version discussed here is also available for download [34]). Species-specific anatomy ontologies for model organism species have a slightly different approach in that they cross-reference VSAO terms and provide formal semantics for the meaning of these cross-references. Thus these databases do not need to use external identifiers. For example, the *Xenopus* Anatomy Ontology (XAO) [35] cross-references VSAO terms; XAO: ‘dermal bone’ is cross-referenced to the vertebrate VSAO: ‘dermal bone’. The semantic meaning of the cross-references is specified in the OBO file header, in this case the frog *Xenopus* ‘dermal bone’ *is_a* VSAO: ‘dermal bone’ that is *part_of* an organism of the taxon *Xenopus*. Although ideally all anatomy ontologies would directly import or include external ontology terms using the MIREOT strategy [36], model organism ontologies have long been in development, and thus updating databases to read external identifiers is too time-intensive. Furthermore, the Uberon, which will incorporate the logical structure and content of the VSAO, cross-references all other anatomy ontologies. Thus, databases pertaining to vertebrate morphology can be queried using VSAO terms.

## Results

### 1. Analysis of Existing Anatomy Ontologies

To build a common representation of skeletal anatomy, we surveyed existing representations in the vertebrate subgroup ontologies (Table 1) to determine the various ways that each had classified skeletal elements and to leverage existing work. Some of the most common issues, including varied representations, found in our examination of the anatomy ontologies were as follows: 1) The representation of bone as an organ, i.e., a skeletal element, and bone as a tissue were conflated as was cartilage as an organ and cartilage as a tissue. In the amphibian (AAO), teleost fish (TAO), the frog *Xenopus* (XAO), and zebrafish (ZFA) anatomy ontologies, for example, the single class ‘bone’ was a type of tissue and was used to classify skeletal elements rather than tissue types. 2) The upper-level skeletal classifications did not relate the multiple organizational levels of the skeletal system to each other. For example, ‘osteocyte,’ a cell type that produces mineralized bone matrix within bone tissue, was not related to ‘bone tissue’ in any of the vertebrate anatomy ontologies. 3) Developmental processes of the skeleton were poorly represented. Many skeletal terms can be defined biologically by the developmental processes producing them, but this was not reflected in the existing anatomy ontologies. For example, endochondral bones were not formally related to the process whereby bone tissue replaces cartilage tissue other than by the fact that they are called endochondral, which presumes the process of endochondral ossification. 4) The multiple relationships to composition and developmental differentia were not well or consistently represented across the ontologies. For example, ‘cartilage element’ *has_part* ‘cartilage tissue’ and ‘cartilage element’ *develops_from* ‘chondrogenic condensation’ were not asserted in any of the vertebrate ontologies.

Following the analysis of existing anatomy ontologies and skeletal classification schemes, we began development of the VSAO by focusing on the properties of skeletal anatomical entities. We used CARO as the upper ontology from which to subclass the VSAO terms. CARO provides a high level classification of anatomical entities, such as cells, tissues, and organs, to link together the different levels of anatomical granularity. Because it is also used by many of the existing anatomy ontologies, it was a natural choice as an upper ontology for the VSAO. We evaluated the Cell Ontology (CL) as a source of cell types from which to link the VSAO. We added new skeletal cell types to it and redefined existing types as appropriate (see section 2.1). To represent the processes involved in skeletal system development, we used terms from the GO Biological Process ontology. For example, VSAO terms are related to GO terms for skeletal development processes (e.g., VSAO: ‘endochondral element’ *participates_in* GO: ‘endochondral ossification’). We proposed six new GO terms that were subsequently added to the GO (‘direct ossification’, ‘intratendinous ossification’, ‘ligamentous ossification’, ‘metaplastic ossification’, ‘perichondral ossification’, and ‘replacement ossification’), and we provided improvements to definitions for others (‘endochondral ossification’, ‘intramembranous ossification’, ‘ossification involved in bone remodeling’, and ‘osteoblast differentiation’). Several existing multispecies anatomy ontologies also contain skeletal types. These include Uberon [29], which has a broader focus in representing structures in all anatomical systems for metazoans, and the Vertebrate Homologous Organ Groups ontology (vHOG) [37], which contains terms based on homologous organ groupings. Future incorporation of the VSAO and vHOG into Uberon will provide an integrated representation of skeletal anatomy for vertebrates across ontologies.

### 2. Classifying Skeletal Anatomy According to Multiple Criteria

**Figure 1:**
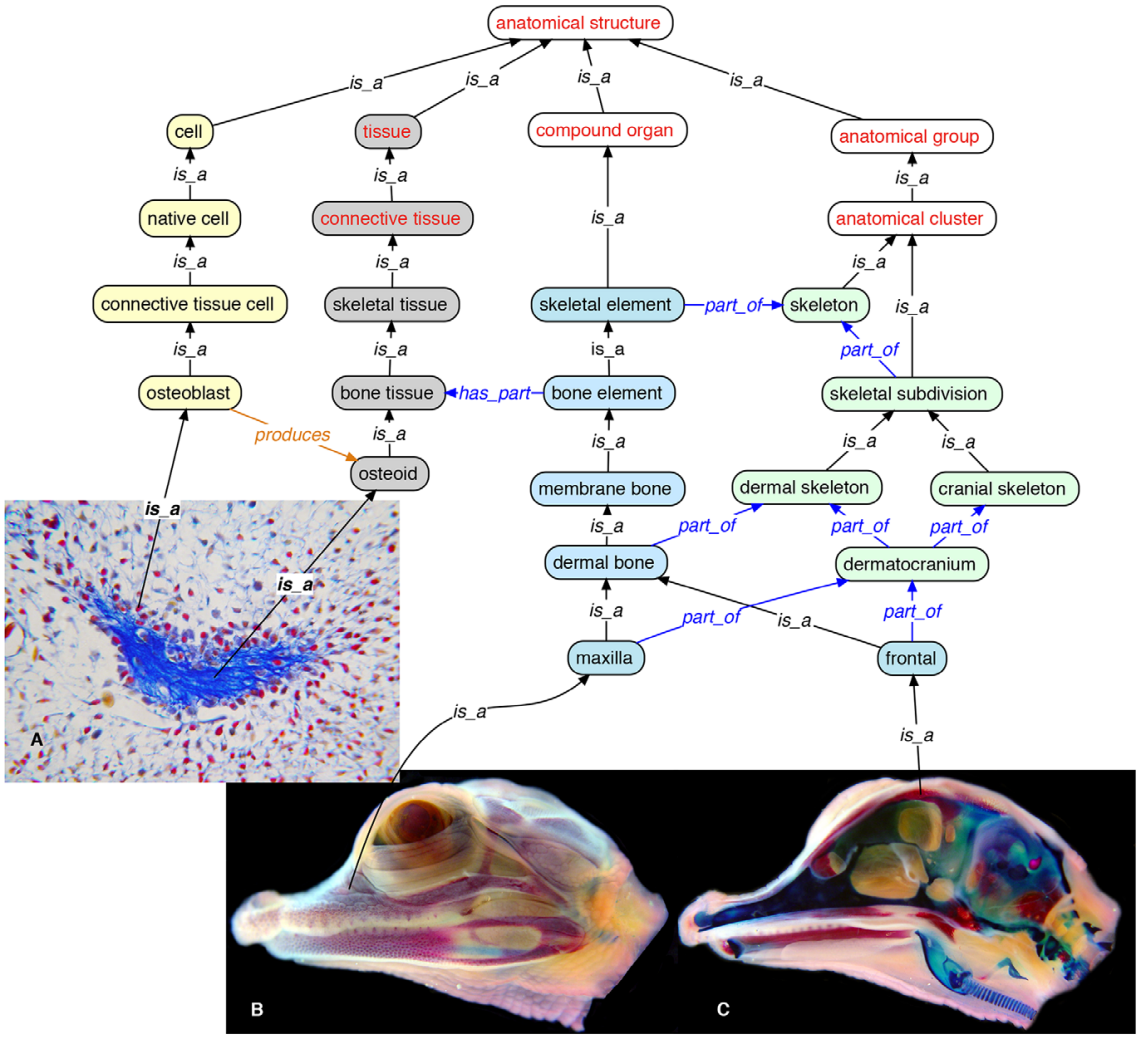
Four high-level classes of skeletal anatomy (‘cell’, ‘skeletal tissue’, ‘skeletal element’, ‘skeletal subdivision’) and their children based on anatomical granularity. Cell terms (CL) are shown in yellow fill, tissue terms in grey fill, skeletal element terms in blue fill, and skeletal subdivision terms in green fill. Parent classes from CARO in red font. *Alligator mississippiensis* sectioned maxilla (∼day 27 in ovo; Ferguson stage 19) stained with Mallory's trichrome (A); midsagittally sectioned embryonic head (day 45 in ovo; Ferguson stage 23) in lateral (B) and saggital (C) view, double stained whole-mount (alizarin red and alcian blue).

In developing the VSAO, we focused on enumerating the essential characteristics (e.g., composition, structure, development) of the components of the skeletal system (e.g., cells, tissues, structures). To avoid errors and omissions (see below and Methods), we automated the task of classification (computing inferred subclass relationships) for bone and cartilage terms by using the OBO-Edit reasoner. We first partitioned skeletal anatomy into four categories based on level of anatomical granularity, from cell types up to organism parts, and made these child concepts of CARO classes (Figure 1). These categories were ‘cell’, ‘skeletal tissue’, ‘skeletal element’, and ‘skeletal subdivision’. We then classified terms based on several axes of classification, reflecting the different ways that biologists describe anatomy, including cell and/or tissue composition, structure, position, biological process, function, and development.

#### 2.1 Cells of the skeletal system

**Figure 2:**
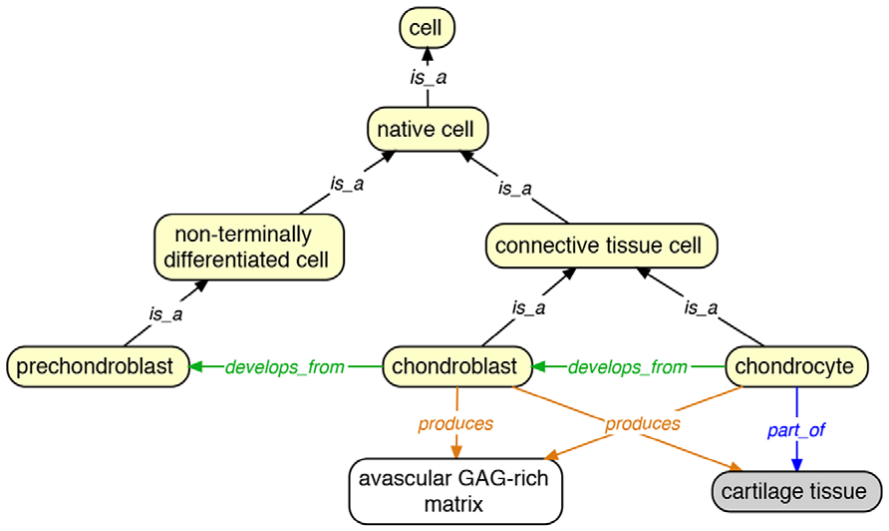
Some skeletogenic cells and their relationships to other cells and skeletal tissues. CL terms are shown in yellow fill, VSAO tissue terms in grey fill.

Accurate representation of cell types is important to define skeletal tissue types, especially where intermediate tissue types are concerned. To enable cross-species inquiry regarding cell type contributions to skeletal development, differences in gene expression, and phenotypic diversity, we related terms in the VSAO to cell terms from the CL. However, for applicability across vertebrates and to relate cells to tissue types, we broadened existing cell term definitions. We also added both new cell types and new developmental relations between new and existing cell types to represent the full diversity of cell types across vertebrates and developmental stages. In the CL, we proposed new definitions for 13 existing skeletogenic cell types, proposed 18 new cell types along with definitions (e.g., ‘skeletogenic cell’, ‘chordoblast’, and ‘preameloblast’), and made eight relationships to specific tissue types. For example, the definition of ‘chondroblast’ in CL was formerly “An immature cartilage-producing cell found in growing cartilage.” Based on our agreed-upon logical differentiae for this cell type, we refined the definition to read “Skeletogenic cell that is typically non-terminally differentiated, secretes an avascular, GAG rich matrix; is not buried in cartilage tissue matrix, retains the ability to divide, located adjacent to cartilage tissue (including within the perichondrium), and develops from prechondroblast (and thus prechondrogenic) cell.” We added relationships from cells to other cells, cellular condensations, and skeletal tissues based on their composition, location, development, and histology (Figure 2), for example:

‘chondroblast’ *is_a* ‘connective tissue cell’.

‘chondroblast’ *develops_from* some ‘prechondroblast’.

‘chondroblast’ *produces* some ‘cartilage tissue’.

‘chondroblast’ *produces* some ‘avascular GAG-rich matrix’.

Logically, these relationships extend to every individual cell of a cell type; for example, every chondroblast produces some cartilage tissue. It is important to note that these logically specified relations allow computation across different levels of granularity and via different axes of classification. This was our central motivation for developing an ontology.

#### 2.2 Skeletal tissue

*‘skeletal tissue’: A specialized form of connective tissue in which the extracellular matrix is firm, providing the tissue with resilience, and/or mineralized and that functions in mechanical and structural support.*

**Figure 3:**
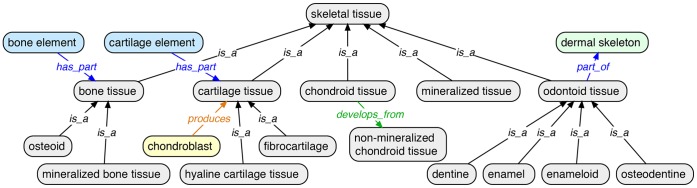
Some skeletal tissues in the VSAO and selected relationships to other tissues, cells, and skeletal elements. CL terms are shown in yellow fill, tissue terms in grey fill, skeletal element terms in blue fill, and skeletal subdivision terms in green fill.

**Figure 4:**
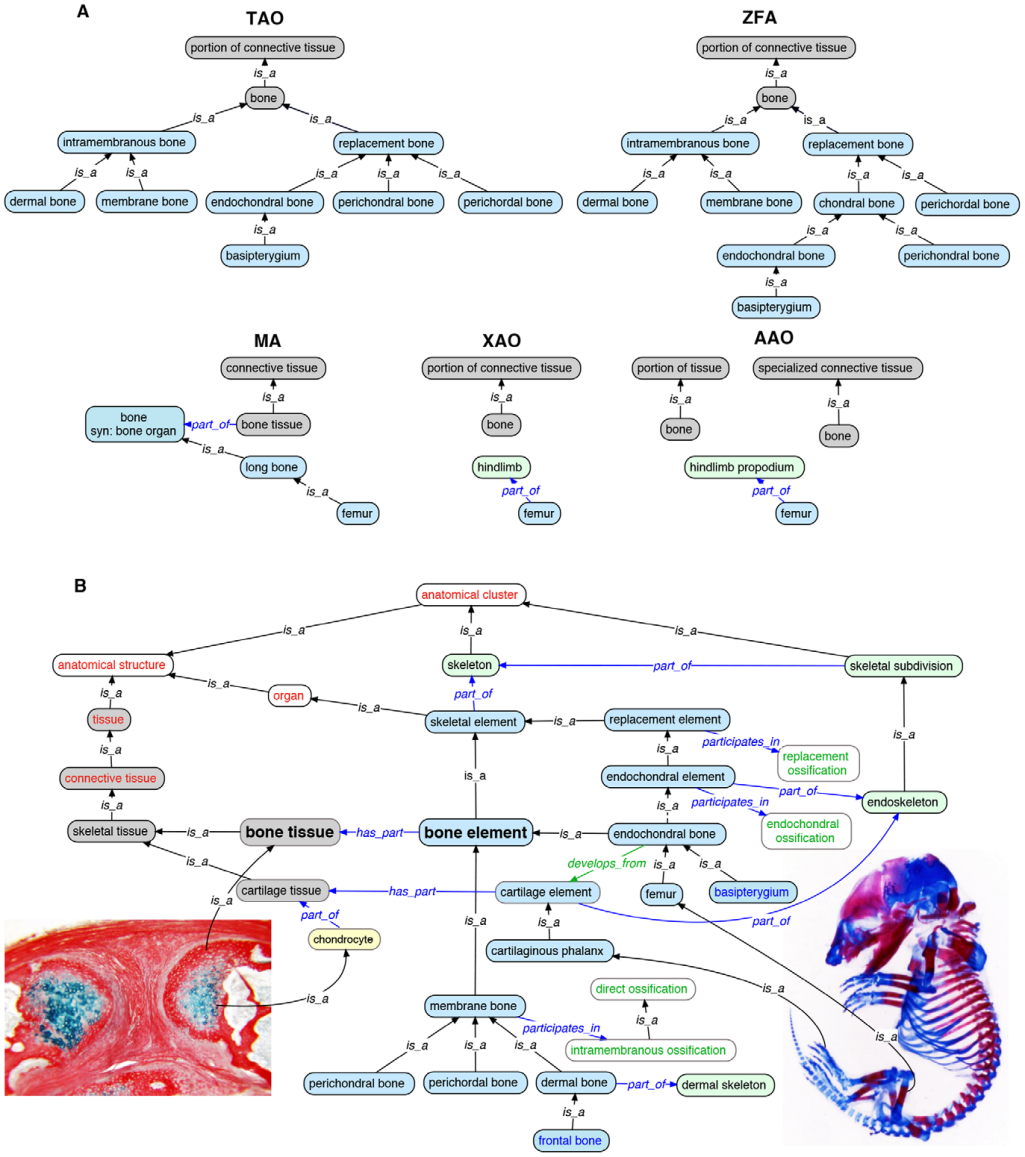
Representation of the skeleton in vertebrate anatomy ontologies. The vertebrate skeleton can be partitioned according to many different criteria – and it had been by the different groups (Table 1) that developed anatomy ontologies. For example (**A**), ‘bone’ had been treated as a type of tissue by all except the MA, who also related it to the concept of ‘bone organ’. In the VSAO (**B**), the concepts of bone tissue and bone element were disentangled, named and defined. Individual bone elements were related to their tissue and cell components as well as developmental processes. From these links one can reason that, e.g., the ‘femur’ is *part_of* ‘endoskeleton’, *develops_from* ‘cartilage element’, and *participates_in* the process of ‘endochondral ossification’, whereas the ‘frontal bone’ is *part_of* ‘dermal skeleton’ and *participates_in* the process of ‘direct ossification’. Image on left shows chondrocytes embedded in a bone matrix developed from periosteum of fractured chick dermal bone. Image on right shows a late gestational stage mouse embryo stained with alcian blue and alizarin red. CL term is shown in yellow fill, tissue terms in grey fill, skeletal element terms in blue fill, and skeletal subdivision terms in green fill. Parent classes from CARO are in red font, GO terms in green font, TAO terms in blue font, and VSAO terms in black font.

Although all of the vertebrate anatomy ontologies recognized some skeletal tissues as tissues, such as ‘bone tissue’ and ‘cartilage tissue’, other tissues were categorized incorrectly. Specifically, enamel and dentine were types of ‘portion of organism substance’ in ZFA and TAO, ‘portion of body substance’ in the human Foundational Model of Anatomy ontology (FMA) [38], and ‘body fluid or substance’ in the MA. Enamel and dentine, and related intermediate tissues such as enameloid and osteodentine, however, are skeletal tissues [39] and we added these to the VSAO as subtypes of ‘odontoid tissue’ (Figure 3). The component vertebrate anatomy ontologies (AAO, TAO, XAO, ZFA) also classified ‘cartilage’ and ‘bone’ as subtypes of ‘connective tissue’ (Figure 4a). To correct this, ‘cartilage element’ and ‘cartilage tissue’ are now separate terms in the VSAO, and subtypes of ‘cartilage tissue’ now include tissue types such as ‘hyaline cartilage tissue’, ‘fibrocartilage’, and ‘secondary cartilage tissue’ (Figure 3). Other newly added types of skeletal tissue in the VSAO include ‘mineralized tissue’, ‘odontoid tissue’, and intermediate tissues such as ‘chondroid tissue’ (Figure 3). The characteristics that distinguish these tissue types has been outlined [40], and this is represented in the VSAO’s tissue hierarchy (see section 2.3 and Figure 3). As described above, although tissues are often defined by their constituent cell types they can also be defined in terms of the extracellular materials they secrete, the developmental processes in which they participate, and the skeletal elements that they comprise.

Skeletal tissue types not universal to vertebrates can be connected to the VSAO through taxon-specific anatomy ontologies. For example, the human anatomy ontology (FMA) includes ‘acellular cementum’ which is present only in mammals and crocodiles [40]. As a type of odontoid tissue, it could be linked to the VSAO in the future within a broader scope ontology such as the Uberon.

#### 2.3 Skeletal elements

*‘skeletal element’: Organ entity that is typically involved in mechanical support and may have different skeletal tissue compositions at different stages.*

‘Bone’ is the most common concept associated with the skeletal system. However, in common usage, this term may refer to either a vertebrate tissue type (bone tissue) or an individuated skeletal element such as the frontal bone. Likewise, in anatomy ontologies, skeletal elements have been represented as types of organs or, incorrectly, as types of tissues. For example, the AAO, TAO, XAO, and ZFA classified ‘bone’ as a type of ‘tissue’ (Figure 4a). The FMA and MA, however, distinguished between ‘bone tissue’ and ‘bone organ’. Similar to conflation of different concepts of bone, most vertebrate ontologies failed to distinguish cartilage tissue from cartilage elements.

VSAO contains the term ‘skeletal element’, which is used in the comparative literature to refer to individual bone or cartilage elements. Individual bones and cartilages are classified in VSAO as ‘skeletal elements’, which are types of ‘organ’ in CARO. We further created the class ‘cartilage element’ for skeletal elements that are composed of ‘cartilage tissue’ and ‘bone element’ for skeletal elements composed of ‘bone tissue’. The crucial part of the CARO definition for ‘organ’ (CARO: ‘compound organ’) is that they are distinct structural units demarcated by bona fide boundaries. By distinguishing bone elements from bone tissues there is flexibility to represent the variety of tissue compositions of different elements in the VSAO. VSAO includes terms for a few skeletal elements that are common to all vertebrates, for example, ‘vertebral element’ [41]. Other individual skeletal element terms (e.g., ‘anocleithrum’) can be linked to VSAO terms based on research requirements.

Skeletal elements have *part_of* relationships to skeletal subdivisions (see 2.4 below) that are based on position. Parthood relationships are used in logical definitions to infer classification based on skeletal subdivisions. For example, ‘cartilage element’ is logically defined based on its *part_of* relationship to the ‘endoskeleton’.

Bone elements are classified according to developmental mode. ‘Membrane bone’ and its subtype ‘dermal bone’ both *participates_in* ‘intramembranous ossification’. ‘Endochondral bone’ has the inferred relationship *participates_in* ‘endochondral ossification’, a relationship inherited from its parent ‘endochondral element’. Teleost ‘frontal bone’ is a subtype of ‘dermal bone’, and from the ontology we can reason that it *participates_in* ‘intramembranous ossification’ (Figure 4b). By articulating these aspects of skeletal elements in relationships between terms, rather than only in a definition of a term, we gain the power to reason across both anatomy and processes for inquiries related to skeletal phenotypes.

#### 2.4 Skeletal subdivisions

*‘skeletal subdivision’: Anatomical cluster consisting of the skeletal elements that are part of the skeleton.*

**Figure 5:**
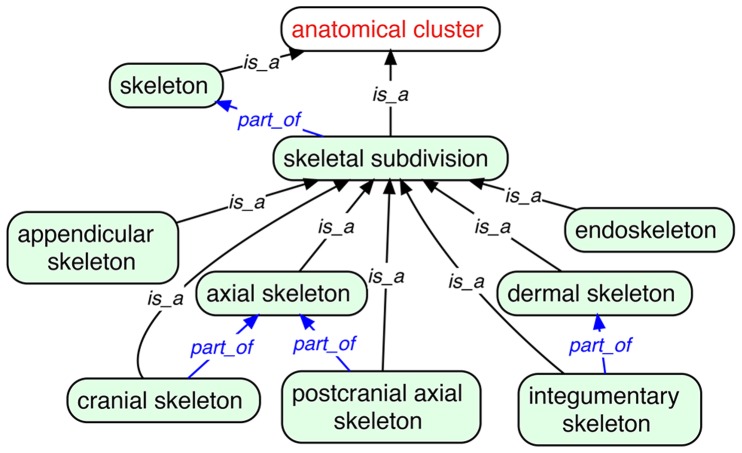
Some skeletal subdivisions and their relationships in the VSAO. CARO parent term is in red font and VSAO terms in black font.

Skeletal subdivisions in the VSAO include the organizational regions ‘appendicular skeleton’, ‘axial skeleton’, ‘cranial skeleton’, ‘integumentary skeleton’, and ‘postcranial axial skeleton’ (Figure 5). The VSAO also contains skeletal subdivision terms based on developmental origin, such as ‘dermal skeleton’, which is defined based on its component entities developing through direct ossification, or the ‘endoskeleton’, which is defined as: “Skeletal subdivision that undergoes indirect development and includes elements that develop as a replacement or substitution of other elements or tissues”.

Just as definitions of skeletal elements may not apply to all vertebrates, the set of skeletal elements that comprise a skeletal subdivision may differ among vertebrate taxa because of evolutionary changes in the development of the skeleton or because of differences in definition across different domains of biological knowledge. The endoskeleton, for example, includes cranial bones such as the intercalar; in teleost fishes, however, the intercalar does not develop from a cartilage precursor [42] but instead develops directly within a connective tissue membrane. Representing the intercalar in the VSAO as *part_of* the endoskeleton would not be appropriate because the *part_of* relationship must hold universally across all taxa. Although this taxonomically variable relationship could be directly specified in individual multispecies or single species anatomy ontologies, there are unlikely to be separate anatomy ontologies for all the taxa of concern. Because VSAO does not describe the taxonomic distribution of anatomy, one way that this variation could be represented is by creating post-compositions of an anatomy term with terms from a taxonomy ontology [12].

### 3. Logical definitions and automating term classification

**Figure 6:**
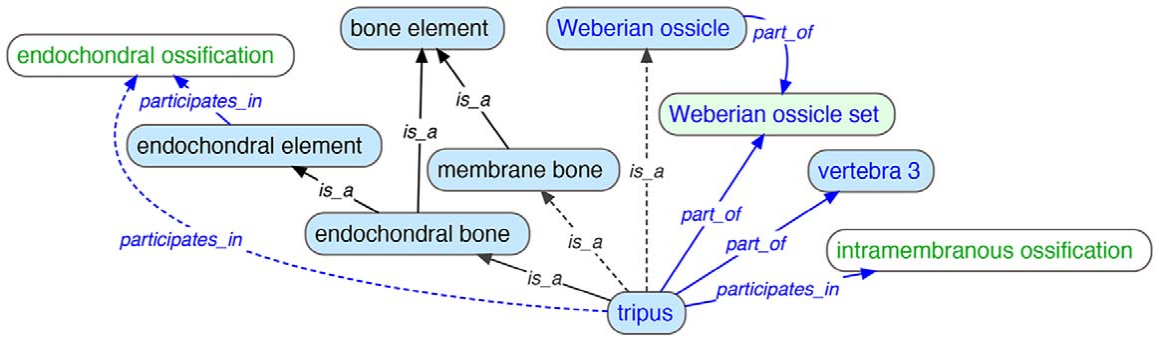
Representation of a skeletal element with multiple classification criteria. The ‘tripus’ is directly asserted (solid lines) to be a type of ‘endochondral bone’, *part_of* the ‘Weberian ossicle set’, *part_of* ‘vertebra 3′ and to form through the process of (‘*participates_in*’) ‘intramembranous ossification’. The reasoner infers (dotted lines) the tripus to be a type of ‘membrane bone’ and a ‘Weberian ossicle’, and infers it to participate in ‘endochondral ossification’. Skeletal element terms are shown in blue fill, skeletal subdivision term in green fill, TAO terms in blue font, VSAO terms in black font, and GO process terms in green font.

Most of the skeletal branches of the various vertebrate anatomy ontologies (Table 1) contained some level of asserted multiple inheritance. Asserted multiple inheritance, in which a term has more than one *is_a* parent (superclass) asserted, can be difficult to maintain in an ontology and can lead both to errors in reasoning [8] and to errors whereby not all children adhere to their parental definitions. Often, however, multiple *is_a* parents reflect a need for biologists to classify entities along multiple conceptual axes. For example, a bone may exhibit two different modes of development within the same organism, as in the tripus, a bone of the axial skeleton in otophysan fishes that develops by both endochondral and intramembranous ossification. ‘Tripus’ would therefore be classified as both a type of ‘endochondral bone’ and ‘membrane bone’ (Figure 6). Similarly, a structure can be classified according to both its developmental and structural attributes. For example, ‘tripus’ is also a type of ‘Weberian ossicle’ because it is a skeletal element that is associated with the Weberian apparatus. Because of these relationships, one could search for the tripus by querying for the structures that *participates_in* ‘endochondral ossification’ or ‘intramembranous ossification’.

A logically preferable way to accommodate multiple inheritances is to infer the polyhierarchy by using logical definitions in which terms are defined by relationships to other terms such that their classification can be automated by a reasoner. A reasoner is a software tool that computationally infers relationships implied by those asserted, including class subsumption relationships. The logical definition of a class constitutes the necessary and sufficient conditions for class membership. In the VSAO, these are of the form ‘An X is a G that D’, where X is the defined class, G is its asserted superclass and D is the set of discriminating characteristic(s) that distinguishes instances of X from instances of other subclasses of G [8], [9]. In the tripus example (Figure 6), rather than subclassify ‘tripus’ with three asserted *is_a* relationships to ‘endochondral bone’, ‘membrane bone’, and ‘Weberian ossicle’, we created logical definitions based on relationships to other terms (*part_of* ‘Weberian ossicle set’, *part_of* ‘vertebra 3′, *participates_in* ‘intramembranous ossification’; Figure 6). Based on these differentiae the reasoner added two implied *is_a* links (*is_a* ‘membrane bone’ and *is_a* ‘Weberian ossicle’). In VSAO, we created logical definitions for types of skeletal elements, which enables multiple classification schemes to be represented in VSAO via reasoning.

Alternatives to creating logical definitions include explicitly naming parts of elements according to development, such as ‘endochondral part of tripus’. This has the disadvantage of introducing terms in the ontology that are unfamiliar to users. A similar but yet more complex scheme could have been adopted for bones composed of multiple developmental types. For example, a class of bone could be introduced such as ‘mixed endochondral/intramembranous bone’ or ‘compound bone’ that would be the single parent for tripus. We decided not to use this scheme because we anticipate that users will search primarily on single developmental types rather than on a combined term.

## Discussion

The VSAO, an expert-vetted skeletal ontology, has the potential to unify the skeletal terminology for species-specific and multispecies anatomy ontologies for vertebrates, and will provide a new level of interoperability and reasoning across fields related to vertebrate anatomy. Previous deficits in comparable terms prevented even simple queries across the databases that house information related to anatomy terms in the various vertebrate component ontologies. For example, a query for ‘bone’ across the vertebrate anatomy ontologies would have produced incomplete or inconsistent results, because ‘bone’ was either represented as a tissue type, or as a skeletal element. Now in the VSAO, ‘bone’ is a synonym for both ‘bone tissue’ and ‘bone element’, and a user would be required to select one or the other for searching. A query using the term ‘bone tissue’ will return skeletal tissues that are subtypes of bone tissue (‘osteoid’ and ‘mineralized bone tissue’), and a query on ‘bone element’ will return all skeletal elements that are composed of bone tissue (subtypes ‘endochondral bone’ and ‘membrane bone’). This will bring clarity to both phenotypic data annotation and to users’ interactions with comparative databases of organismal phenotypes.

The new sets of rich connections from skeletal elements in VSAO to tissues, cell types (via CL), and developmental processes (via GO), support more sophisticated queries than were possible before. The following examples illustrate the kinds of questions that can be facilitated with the use of this skeletal anatomy module, provided its integration with a full set of anatomical concepts, a collection of phenotype annotations to taxon concepts, and a reasoner that infers relationships entailed by those asserted:

- Find the cell types that contribute to the development of endochondral bones. In VSAO, ‘endochondral bone’ *develops_from* ‘cartilage element’, and ‘cartilage element’ *has_part* ‘cartilage tissue’, which, in turn, is produced by ‘chondroblast’. Thus, ‘chondroblast’ would be one of the inferred cell types from which endochondral bones develop.
- Find all the integumentary structures (teeth, scales, etc.) that receive extracellular matrix contributions from odontoblasts. In VSAO, ‘odontoblast’ *produces* ‘dentine’. Hence, any structures asserted or inferred to have dentine tissue as a part would be found in such a query.
- Find all the skeletal elements across vertebrates that develop, at least in part, via intramembranous ossification. VSAO asserts that ‘membrane bone’ *participates_in* ‘intramembranous ossification’, and therefore this query would result in all skeletal elements that are subtypes of ‘membrane bone’, or that contain a membrane bone part. Because the species-specific databases contain skeletal phenotypes annotated to genes, this query can be expanded to: ‘find all genes associated with all skeletal elements that participate in intramembranous ossification’. A user, for example, might want to compare this list of genes with a list of genes involved in endochondral ossification to begin to understand patterns of gene regulation and expression in relation to different modes of bone formation.

Homologous cells, tissues, and elements of the skeleton of vertebrates are well known to vary among species in their development, structure, and composition. For example, at the cellular level, osteocytes originate from both mesodermal and neural crest cells [43]. The vertebral centrum is an example of a skeletal element with differences in composition and development not only across taxa, but also within individuals. The vertebral centrum may be cartilaginous (e.g., caudal vertebrae in living dipnoans and young elasmobranchs [44]), develop as cartilage but be replaced by bone (most tetrapods) or become mineralized (elasmobranchs), or form directly through intramembranous ossification (some amphibians and fishes). It is critical to accurately represent these skeletal differences among taxa, and our goal was to create a high-level ontology structure that enables this representation. Thus the term ‘vertebral centrum’ was defined to accommodate all of these types. It is not defined by tissue type or development, but by position and structure. Taxon-specific centrum types defined based on composition or development can be linked to the parent term for vertebral centrum.

Homologous skeletal structures can also vary in their position or location across taxa, sometimes dramatically. The highly derived body plan of turtles, for example, involves the repositioning of the scapula inside the rib cage rather than outside as in other amniotes [45], [46]. Given this taxonomic variation and that the scapula is *part_of* the pectoral girdle, the pectoral girdle is not defined in relation to the rib cage but rather as one in which both dermal and endoskeletal elements connect the anterior appendicular skeleton to the axial or cranial skeleton.

### Future Directions

As new terms are required for the representation of phenotypes from additional vertebrates (e.g. sharks, birds) to meet research needs, the VSAO provides an umbrella under which to add and relate more specific new terms. Integration of the VSAO with the human anatomy ontologies is a challenge for the future. Terminology for human anatomy diverges from that of other vertebrates in many respects [2]. For example, positional terms differ between studies of humans and other vertebrates: the chest and stomach of humans is described as ‘anterior’, in contrast to other vertebrates in which they are described as ‘ventral’. Names of skeletal elements and tissues in humans may also differ from other vertebrates. For example, the term ‘ossicle’ is standardly used in human anatomy to refer to the small jointed bones in the middle ear. Comparative vertebrate anatomists, however, include skeletal elements of variable composition (not only bone) and not necessarily jointed as other examples of ossicles [47]. Ossicles in the VSAO include ‘appendicular ossicle’, ‘axial ossicle’, ‘ossified tendon’, and ‘sesamoid’ (including, e.g., the patella in mammals). Integration with human ontologies, for example, through the Uberon, will facilitate model system and evolutionary biology because, via medical biology, humans are perhaps the most studied vertebrate species.

A major challenge to integration, in addition to the full incorporation of the VSAO in model organism ontologies, will be coordinating term addition and maintaining synchrony with the VSAO over time. Tools to automate this process are currently lacking, and thus maintaining a unified concept of the skeleton relies upon communication across the community of biologists and ontologists.

### Conclusions

The desire of disparate communities to share data across databases and to unify semantically similar concepts motivated the development of the VSAO and its incorporation in taxon-specific ontologies. VSAO is a module of anatomical concepts for the vertebrate skeletal system which unifies the existing terminologies in multi-species and single-species anatomy ontologies. The creation and adoption of this ontological superstructure will enable addressing key research questions, as well as the discovery of new knowledge.
